# Aesthetic nursing and aromatherapy: reflection on interlocution, applicability and potential

**DOI:** 10.1590/0034-7167-2024-0315

**Published:** 2025-08-08

**Authors:** Amanda Karina Santos Vieira, Ruth Natalia Teresa Turrini, Gisele Chicone, Juliana Rizzo Gnatta

**Affiliations:** I Universidade de São Paulo. São Paulo, São Paulo, Brazil

**Keywords:** Esthetics, Nursing, Complementary Therapies, Aromatherapy, Cosmetic Techniques., Estética, Enfermería, Terapias Complementarias, Aromaterapia, Técnicas Cosméticas.

## Abstract

**Objectives:**

to reflect on aromatherapy in the specialty of aesthetic nursing.

**Methods:**

a theoretical essay of a reflective nature, with integrative and complementary health as a conceptual framework, supported by both international and national literature, with analysis of previous and current professional legislation.

**Development:**

presented in two topics that are interconnected and discuss the points of convergence between the topics under analysis: the increased demand for products of natural origin and for aesthetic procedures that are less invasive point to the dialogue, applicability and potential of aromatherapy in aesthetic nursing.

**Final Considerations:**

recent legislation on aesthetic nursing in Brazil indicates the constant improvement of this specialty, which reinforces the need for work that enables the transfer of knowledge for safe and effective practice.

## RESUMEN

**Objetivos:**

reflexionar sobre la aromaterapia en la especialidad de enfermería estética.

**Métodos:**

ensayo teórico de carácter reflexivo, con la salud integrativa y complementaria como marco conceptual, apoyado en la literatura internacional y nacional, con análisis de la legislación profesional pasada y actual.

**Desarrollo:**

se presenta en dos temas interconectados que discuten los puntos de convergencia entre los temas en análisis: la creciente demanda por productos de origen natural y por procedimientos estéticos menos invasivos apuntan al diálogo, aplicabilidad y potencialidad de la aromaterapia en la enfermería estética.

**Consideraciones Finales:**

la legislación reciente sobre enfermería estética en Brasil indica el perfeccionamiento constante de esta especialidad, lo que refuerza la necesidad de trabajos que posibiliten la transferencia de conocimientos para una práctica segura y eficaz.

## INTRODUCTION

The developments for the regulation of aesthetic nursing are recent, with the initial milestone being COFEN Resolution 529/2016^(1)^, which underwent adjustments through COFEN Resolutions 626/2020 and 715/2023, which established the list of permitted aesthetic procedures and the requirement for specialist registration with the professional body of the state of operation^(1)^. In this way, nursing is supported to, among other things, carry out nursing consultations, anamnesis and establish the most appropriate treatment for the person, in addition to creating protocols, prescribing home care and self-care guidelines for those undergoing aesthetic procedures, and performing aesthetic procedures that involve the use of cosmetics and cosmeceuticals^(1)^.

Care in aesthetics is not restricted to procedures or techniques, but is concerned with providing health, well-being, quality of life and self-esteem through comprehensive assistance to individuals^(2)^.

In most aesthetic treatments, the focus is on caring for the skin, the largest organ in the human body, which acts to protect against external damage^(3)^. In this regard, several anti-aging skin strategies have been developed, as well as treatments with resources of plant and mineral origin that, in addition to enabling a more natural appearance, can have a significantly positive impact, due to the implication of synthetic ingredients on human health and the environment^(4)^.

Aromatherapy, a branch of phytotherapy that studies aromatic plants, is historically associated with beauty and well-being^(5)^. The technique is configured as an Integrative and Complementary Health Practice (ICHP), expanding the options for global healthcare for human beings by also considering their integrality.

ICHP, care strategies that are distinct from those of the conventional biomedical model, by incorporating different medical rationalities and traditional knowledge, are added to nursing specialties, according to COFEN Resolution 581/2018^(6)^. Concerning aromatherapy, the legality of the use of this technique by nursing professionals is guaranteed both by Technical Chamber Opinion 34/2020/CTLN/COFEN^(7)^, which legitimizes the prescription of essential oils (EOs) by nurses, and by COFEN Resolution 739/2024^(8)^, which determines a minimum training load of 120 hours for their work as an aromatherapist.

Widely used in the cosmetics industry for the formulation of fragrances, aromatherapy has also been explored for aesthetic treatments, due to the antioxidant potential that some EOs have^(4)^. The relevance of plant-based resources for the formulation of natural products that act on skin anti-aging has been progressively demonstrated, with efficacy observed both for hydration and for repairing the skin’s protective barrier against the effects of ultraviolet radiation and free radicals^(3,9)^.

Currently, several anti-aging actions and techniques are available, such as lifestyle changes, cosmetic care, adequate sun protection and non-invasive and invasive aesthetic procedures. However, consumers in aesthetics have increasingly prioritized their health and well-being, wanting non-invasive, naturally-derived products and treatments that guarantee safety and efficacy^(3)^.

Considering the legal support for nurses’ work in aesthetics, ICHP and aromatherapy, in addition to the potential dialogue between aromatherapy and aesthetics and the growing demand for aesthetic care strategies that are natural and promote health and well-being, this study is based on the central question: how can aromatherapy be used in the context of the aesthetic nursing specialty?

## OBJECTIVES

To reflect on aromatherapy in the specialty of aesthetic nursing.

## METHODS

Theoretical essay of a reflective nature, whose methodological principle is the idea of not worrying too much about achieving a new feat or creating something, but rather exploring and considering both the positive and negative aspects that surround one or more existing conceptions^(10)^. Integrative and complementary health was used as a conceptual framework, which involves individuals’ health as a whole, considering the physical, emotional, mental, social and spiritual aspects to promote well-being with a person-centered focus.

The motivation for this work is due both to the authors’ experience with ICHP and aesthetic nursing and to the growing global concern with sustainability in terms of the human-environment relationship and the generation of synthetic waste, and with integrative health - care for the mind, body and spirit.

Reflective analysis was based on international and national literature; the latter was carried out based on previous and current Brazilian nursing legislation regarding ICHP, aromatherapy and aesthetics.

The selection of bibliographical references was made with the aim of supporting the reader so that, when coming into contact with the Brazilian laws that regulate ICHP and aesthetic nursing, with the scope of action of aesthetic nursing professionals and, even briefly, with the emergence of aromatherapy and its historical relationship with beauty and aesthetics, they can weave their own perceptions based on what will be raised throughout the text.

## DEVELOPMENT

### Aesthetics and nursing

The concept of beauty spans the history of humanity and changes over time due to cultural and religious values, politics and region^(11)^. Although it may be associated with observers’ subjective feelings, the definition of beauty is strongly disseminated by the mainstream media following the trend of globalization and a universal standard of beauty, in disparity with the singularities of subjects and the diverse cultures existing in the world^(11)^.

Slim bodies, symmetrical facial features and smooth skin, without acne, without visible pores, well hydrated and flushed, are considered fundamental points for individuals to be able to meet the idealized and imposed standard^(11)^. However, there is a growing effort to break down these labels so that there are no obstacles for anyone to be seen or perceive themselves as beautiful, regardless of their imperfections, body sizes and skin colors^(11)^.

The constant concern with meeting beauty ideals not only increases the demand for aesthetic procedures, but also generates exacerbated levels of dissatisfaction, which can lead to mental illness, with the development of Body Dysmorphic Disorder, a condition in which individuals are incessantly concerned with small, sometimes non-existent, problems in relation to their appearance^(11)^.

Despite the numerous aesthetic procedures that not only encourage this type of condition, but are also extremely invasive, there is a tendency for aesthetics to be increasingly associated with promoting health, generating self-esteem and well-being, quality of life, and balance of body and mind.

By understanding aesthetics as a specialty to be performed by healthcare professionals, their work objective could go beyond the construction or improvement of what is or is not beautiful. In line with the promotion of well-being, quality of life and self-esteem - aspects intrinsic to health - it would be possible to establish a gradual distancing from the insistent association that the global media makes of aesthetic health with a sickening standard of universal beauty.

Accordingly, the question is raised about the possibility of alternative and/or complementary care favoring an integrative vision of aesthetics, in order to provide personal satisfaction, beyond the incessant concern with being pleasing in the eyes of others.

From the perspective of integrative health, these issues invite us to think about the role of nursing professionals, the largest professional category in the health area in Brazil, in ensuring this care. Since they provide comprehensive care to human beings, seeing them as biopsychosocial-spiritual beings, aesthetic nurses are able to indicate and guide aesthetic treatments and the physical and emotional care that involves them, ensuring humanization and the necessary support to individuals^(2)^.

In their training, nurses develop anatomical knowledge of the skin and tissue repair, with mastery of techniques, skills and abilities for comprehensive care of subjects, through communication, leadership, decision-making, and management of situations and people, in a critical and reflective way^(2)^.

Regarding nursing legislation within the aesthetic specialty, it was the aforementioned COFEN Resolution 529/2016 that paved the way, by making aesthetic nurses qualified to perform carboxytherapy, use of cosmetics and cosmeceuticals, dermopigmentation, lymphatic drainage, electrotherapy/electrothermophototherapy, combined ultrasound and microcurrent therapy, micropigmentation, cavitational ultrasound, and vacuum therapy^(1)^.

Not infrequently, new interventions generate conflicts in the limits of action among healthcare professionals, and amid public court orders, mobilization of nurses in scientific events and debates for professional recognition in the area, COFEN Resolution 529/2016 was amended by Resolution 626/2020, which ensures the performance of specialist nurses in the area, respecting the non-execution of procedures strict to medical professionals^(1)^.

Due to scientific and technological advances and demands from aesthetic nurses, Technical Chamber Opinion 001/2022/GTEE/COFEN added more techniques to the range of those already permitted, such as platelet-rich plasma, intramuscular application of botulinum toxin, endermotherapy, facial harmonization and other injectable techniques, in addition to the application of polydioxanone support threads for biostimulation through cannulas and percutaneous induction of active ingredients and dermal fillers^(2)^.

Subsequently, Resolution 715/2023 detailed the rigor for the legitimacy of the specialty, whose *lato sensu* graduate degree, in addition to being duly recognized by the Ministry of Education and Culture, must have a minimum of 100 hours of supervised practical activities^(1)^.

That said, given so much technicality, would there be room for a practice that, in addition to providing care for appearance, would seek to look at individuals beyond their physical body, in order to provide healthier, less stigmatizing and more comprehensive aesthetic care? Interventions that use resources of natural origin and that guarantee ease of access, use and long-term effects, together with the movement to expand ICHP for health promotion through the perspective of integrative health, with a holistic approach to individuals, place aromatherapy as a viable alternative for aesthetic treatments that seek to integrate health with beauty care.

### Aromatherapy and aesthetics

The term “aromatherapy” was coined in 1935 by René Maurice Gattefossé. In 1937, Gattefossé published the book “*Aromathérapie*”, which disseminated vast scientific knowledge about the relationships between the properties of the molecules present in the most diverse EOs, placing France as a country with a strong influence in the history of scientific aromatherapy^(5)^. In England, inspired by Gattefossé, nurse Marguerite Maury began working on aromatherapy applied to beauty, youth and well-being care^(5)^, and in 1961, she published the book “*Le Capital Jeunesse*”. The expansion in the use of aromatherapy led to its inclusion in the International Classification of Diseases 11^th^ revision in chapter X - Extension codes, in the personal use item - XE4KX Essential oils, oils used in aromatherapy.

With the aim of providing skin care, improving its appearance and appearance, aesthetic formulations aim to prevent and/or treat the effects of aging, with active ingredients that delay or even reverse environmental and age-related damage. In view of this, studies point to the potential of natural active ingredients in combating skin aging, repairing damage to elastin, acting against free radicals and photoaging, and improving skin hydration, regeneration and nutrition^(3,4,9)^.

Plant-based resources have the advantage of being considered more reliable and safe, with a gentle and effective action, when compared to synthetic molecules^(9)^, and have been used for centuries for skin care purposes. In India, China, Egypt and Iran, the use of plants for cosmetic purposes has been confirmed for thousands of years^(4)^. Currently, there is a progressive increase in the demand for cosmetics with sustainable production and consumption patterns, and by 2021, around 40 billion dollars had already been spent on cosmetic products of natural origin, around 10% of the global market in the sector^(4)^.

In this scenario, there are low molecular weight volatile compounds from the most diverse parts of plants (flowers, leaves, branches, seeds, stems, fruits and roots), formed by a mixture of organic substances, such as terpenic hydrocarbons, alcohols (e.g., geraniol), aldehydes (e.g., citronellal), ketones and esters^(9)^. Like other plant resources, they have also been used for aesthetic purposes for thousands of years and, today, are widely relevant in the pharmaceutical, agricultural, food, cosmetic and health industries^(3,4,9)^.

Antimicrobial and antioxidant properties, which combat oxidative stress and free radicals^(3,4,9)^, guarantee the presence of EOs in several innovative formulations for skin care, cleansing and protection^(9)^, increasingly replacing synthetic antioxidants, which have toxic effects on the body^(4)^ and are harmful to the environment.

Organic compounds such as geraniol and citronellal, found in geranium (*Pelargonium graveolens*) and damask rose (*Rosa x damascena*) oils, act to eliminate these free radicals and reduce oxidative stress^(5,8)^, which makes them promising allies in the fight against skin aging. Furthermore, oils with different biochemical compositions, such as rosewood (*Aniba roseadora*) and thyme (*Thymus vulgaris qt linalool*), also have anti-skin aging properties, by reducing wrinkles and fine lines^(5)^.

Based on these data, it is appropriate to reflect on the interlocution, applicability and potential of aromatherapy in the specialty of aesthetic nursing: interlocution, due to the fact that both encompass care with effects that result in well-being for individuals undergoing treatment; applicability, due to the numerous properties (of EOs): antiseptic (*Cinnamomum verum*), anti-inflammatory (*Lavandula angustifolia*), analgesic (*Mentha x piperita*) and antioxidant (*Pelargonium graveolens*)^(5)^, which would allow, for instance, the topical use at home of formulations containing EOs to maximize the effects of the aesthetic intervention performed in the office, or the concomitant use of an EO with analgesic action to perform a painful aesthetic procedure; and potential, due to the use of aromatherapy as an integrative and complementary practice to usual aesthetic procedures, or even respecting individuals’ preferences as an isolated technique for more natural intervention in aesthetics.

Finding solutions to treat signs of skin aging has been a human desire for centuries, and the beauty market is full of resources for this purpose. Furthermore, aromatherapy fits perfectly into the sustainable cosmetics policy.

Within this scenario, the increase in demand for products of natural origin, with less impact on bodily health and the environment, as well as for aesthetic procedures that are less invasive, indicates a growing axis of convergence between aromatherapy and aesthetics, even though the accentuated sense of urgency with which we live and have become accustomed makes it difficult to accept techniques that, being natural and non-invasive, may take longer to guarantee results.

## FINAL CONSIDERATIONS

Based on the reflections made, it seems appropriate to conclude by encouraging scientific productions that seek to analyze the interconnection of these two areas, in order to expand the offer of aesthetic treatments to the interested population and ensure that aesthetic nursing professionals have more and more support to work in the area. Recent legislation on aesthetic nursing in Brazil shows us that the specialty is in evidence and constantly developing, which reinforces the need for studies that enable the transfer of knowledge for safe and effective practice.

The reflective nature of this work stands out, as it seeks to weave provocations around less invasive natural interventions for aesthetic care, since, immersed in a society full of stigmatizing and sickening standards, and which consumes and generates shocking amounts of synthetic waste, harmful to both human health and the environment, it is urgent that we expand the discussion of sustainability even to the aesthetic market and, furthermore, review the way we see - or refute - the ideas of beauty.

However, immersed in an immediate reality, with a frenetic pace and already very taken by the universal beauty standard, inserting into the aesthetics market a practice that, in addition to adding to those who wish to reveal a beauty that is not always so symmetrical, allows associating aesthetic care with holistic care, can be not only challenging for many, but unacceptable for some.

Above all, this work seeks to awaken opportunities in the areas of nursing and health, and in the context of aromatherapy applied to aesthetics, given that, in addition to the expansion of the scope of action of nurses and other healthcare professionals, as already mentioned, the continuous technical advances in the area of aesthetics do not keep up with the accessibility of its offer of procedures for a large part of the population, due to the cost of certain protocols, their durability and the lack of coverage by supplementary health.

## Data Availability

Not applicable.
