# Supplementary material for: Aesthetic nursing and aromatherapy: reflection on interlocution, applicability and potential
Source: Rev Bras Enferm. 2025 Aug 8;78(3):e20240315. doi: 10.1590/0034-7167-2024-0315 (PMC12334204; doi:10.1590/0034-7167-2024-0315)
Supplement: Translation

# Translation: Enfermagem estética e aromaterapia: reflexão sobre a interlocução, a aplicabilidade e a potencialidade

**DOI:** 10.1590/0034-7167-2024-0315pt

**Authors:** Amanda Karina Santos Vieira, Ruth Natalia Teresa Turrini, Gisele Chicone, Juliana Rizzo Gnatta

**Affiliations:** I Universidade de São Paulo. São Paulo, São Paulo, Brasil

**Keywords:** Estética, Enfermagem, Terapias Complementares, Aromaterapia, Técnicas Cosméticas.

## Abstract

**Objetivos:**

refletir acerca da aromaterapia na especialidade de enfermagem estética.

**Métodos:**

ensaio teórico de cunho reflexivo, tendo a saúde integrativa e complementar como marco conceitual, sustentado tanto em literatura internacional quanto nacional, com análise da legislação profissional pregressa e atual.

**Desenvolvimento:**

apresentado em dois tópicos que interligam-se e discorrem sobre os pontos de convergência entre as temáticas em análise: o aumento da procura por produtos de origem natural e por procedimentos estéticos que sejam menos invasivos apontam para a interlocução, a aplicabilidade e a potencialidade da aromaterapia na enfermagem estética.

**Considerações Finais:**

as recentes legislações da enfermagem estética no Brasil indicam o constante aperfeiçoamento dessa especialidade, o que reforça a necessidade de trabalhos que possibilitem a transferência de conhecimentos para uma prática segura e eficaz.

## INTRODUÇÃO

Os desdobramentos para a regulamentação da enfermagem estética são recentes, com marco inicial pela Resolução COFEN nº 529/2016^(1)^, a qual passou por ajustes através das Resoluções COFEN nº 626/2020 e nº 715/2023, que estabeleceram o rol de procedimentos estéticos permitidos e a exigência pelo registro de especialista no órgão profissional do estado de atuação^(1)^. Dessa forma, a enfermagem está respaldada para, entre outros, realizar consulta de enfermagem, anamnese e estabelecer o tratamento mais adequado à pessoa, além de criar protocolos, prescrever cuidados domiciliares e orientações de autocuidado àqueles submetidos aos procedimentos estéticos, e realizar procedimentos estéticos que envolvem o uso de cosméticos e cosmecêuticos^(1)^.

O cuidar na estética não se restringe a procedimentos ou técnicas, mas preocupa-se em proporcionar saúde, bem-estar, qualidade de vida e autoestima através da assistência integral ao indivíduo^(2)^.

Na maioria dos tratamentos estéticos, o foco é o cuidado com a pele, o maior órgão do corpo humano, o qual atua na proteção contra danos externos^(3)^. Nesse sentido, diversas estratégias antienvelhecimento da pele têm sido desenvolvidas, bem como tratamentos com recursos de origem vegetal e mineral que, além de possibilitar uma aparência mais natural, podem ter um impacto significativamente positivo, devido à implicação dos ingredientes sintéticos na saúde humana e no meio ambiente^(4)^.

A aromaterapia, ramo da fitoterapia que estuda as plantas aromáticas, está, historicamente, associada à beleza e ao bem-estar^(5)^. A técnica configura-se como uma Prática Integrativa e Complementar de Saúde (PICS), expandindo as opções de atenção à saúde global do ser humano ao considerar também a sua integralidade.

As PICS, estratégias de cuidado que se distinguem daquelas do modelo biomédico convencional, por incorporarem diferentes racionalidades médicas e conhecimentos tradicionais, somam nas especialidades de enfermagem, conforme a Resolução COFEN nº 581/2018^(6)^. No que concerne à aromaterapia, a legalidade da utilização desta técnica pelo profissional enfermeiro é garantida tanto pelo Parecer de Câmara Técnica nº 34/2020/CTLN/COFEN^(7)^, o qual legitima a prescrição de óleos essenciais (OEs) pelo enfermeiro, quanto pela Resolução COFEN nº 739/2024^(8)^, que determina carga horária mínima de 120 horas de formação para sua atuação como aromaterapeuta.

Bastante utilizada na indústria cosmética para a formulação de fragrâncias, a aromaterapia tem sido explorada também para tratamentos estéticos, devido ao potencial antioxidante que alguns OEs possuem^(4)^. A relevância dos recursos de fontes vegetais para a formulação de produtos naturais que atuem no antienvelhecimento da pele tem sido progressivamente demonstrada, com eficácia observada tanto para a hidratação quanto para a reparação da barreira protetora da pele em relação aos efeitos da radiação ultravioleta e dos radicais livres^(3,9)^.

Atualmente, diversas ações e técnicas antienvelhecimento estão disponíveis, como mudanças no estilo de vida, cuidados cosméticos, proteção solar adequada e procedimentos estéticos não invasivos e invasivos. No entanto, os consumidores em estética têm priorizado cada vez mais sua saúde e bem-estar, desejando produtos e tratamentos não invasivos, de origem natural, que garantam segurança e eficácia^(3)^.

Considerando o respaldo legal para atuação do enfermeiro na estética, nas PICS e na aromaterapia, além da potencial interlocução existente entre a aromaterapia e estética e a crescente procura por estratégias de cuidados em estética que sejam naturais e promovam saúde e bem-estar, este estudo pauta-se na questão central: como a aromaterapia pode ser utilizada no contexto da especialidade de enfermagem estética?

## OBJETIVOS

Refletir acerca da aromaterapia na especialidade de enfermagem estética.

## MÉTODOS

Ensaio teórico de cunho reflexivo, cujo princípio metodológico está na ideia de não se preocupar demais em alcançar um novo feito ou em criar algo, mas sim em explorar e ponderar tanto os aspectos positivos quanto os negativos que circundam uma ou mais concepções já existentes^(10)^. Utilizou-se como marco conceitual a saúde integrativa e complementar que envolve a saúde do indivíduo como um todo, considerando os aspectos físicos, emocionais, mentais, sociais e espirituais para promover o bem-estar com foco centrado na pessoa.

A motivação para este trabalho se deve tanto à experiência dos autores com PICS e enfermagem estética quanto à crescente preocupação mundial com a sustentabilidade no que se refere à relação homem-meio ambiente e à geração de resíduos sintéticos, e com a saúde integrativa - cuidado com a mente, o corpo e o espírito.

A análise reflexiva pautou-se na literatura internacional e nacional; esta última foi feita a partir das legislações pregressas e vigentes da enfermagem brasileira no que concerne às PICS, à aromaterapia e à estética.

A seleção das referências bibliográficas foi feita com o intuito de subsidiar o leitor para que, ao entrar em contato com as leis brasileiras que regulamentam as PICS e a enfermagem estética, com o escopo de atuação do profissional enfermeiro esteta e, ainda que brevemente, com o surgimento da aromaterapia e sua relação histórica com a beleza e a estética, possa tecer suas próprias percepções a partir do que será levantado ao longo do texto.

## DESENVOLVIMENTO

### A estética e a enfermagem

O conceito de beleza atravessa a história da humanidade e muda, ao longo do tempo, em razão dos valores culturais e religiosos, da política e da região^(11)^. Embora possa estar associado aos sentimentos subjetivos do observador, a definição do belo é fortemente difundida pelas grandes mídias seguindo a tendência da globalização e de um padrão de beleza universal, em disparidade às singularidades dos sujeitos e das diversas culturas existentes no mundo^(11)^.

Corpos magros, características faciais simétricas e pele lisa, sem acnes, sem poros visíveis, bem hidratada e corada, são tidos como pontos fundamentais para que o indivíduo possa atender ao padrão idealizado e imposto^(11)^. No entanto, há um crescente esforço em quebrar esses rótulos, a fim de que não haja obstáculos para que alguém seja visto ou se perceba como belo, independentemente de suas imperfeições, tamanhos corporais e cores de pele^(11)^.

A constante preocupação em corresponder aos ideais de beleza não só aumenta a procura por procedimentos estéticos, mas também gera quadros exacerbados de insatisfação, podendo levar ao adoecimento psíquico, com o desenvolvimento de Transtorno Dismórfico Corporal, condição na qual o indivíduo preocupa-se incessantemente com pequenos problemas, às vezes inexistentes, em relação à sua aparência^(11)^.

Apesar dos inúmeros procedimentos estéticos que não só fomentam esse tipo de quadro, mas que também são extremamente invasivos, há a tendência de que a estética esteja cada vez mais associada à promoção da saúde, ao gerar autoestima e bem-estar, qualidade de vida, e equilíbrio do corpo e da mente.

Ao se entender a estética como uma especialidade a ser desempenhada por profissionais de saúde, seu objeto de trabalho poderia ir além da construção ou aprimoramento do que é ou não belo. Na linha da promoção do bem-estar, da qualidade de vida e da autoestima - aspectos intrínsecos à saúde -, seria possível estabelecer um gradual distanciamento da insistente associação que a mídia global faz da saúde estética com um padrão adoecedor de beleza universal.

Nessa lógica, indaga-se sobre a possibilidade de cuidados alternativos e/ou complementares favorecerem uma visão integrativa da estética, de modo a proporcionar satisfação pessoal, para além da incessante preocupação em ser agradável aos olhos de outrem.

Sob a ótica da saúde integrativa, essas indagações nos convidam a pensar sobre o lugar do profissional enfermeiro, maior categoria profissional da área da saúde no Brasil, para a garantia desse cuidado. Por ter, em suas práxis, a atenção integral ao ser humano, enxergando-o como um ser biopsicossocioespiritual, o enfermeiro esteta é capaz de indicar e orientar os tratamentos estéticos e os cuidados físicos e emocionais que envolvem os mesmos, garantindo humanização e suporte necessário ao indivíduo^(2)^.

Em sua formação, o enfermeiro desenvolve conhecimentos anatômicos da pele e reparação tecidual, com domínio de técnicas, competências e habilidades para a atenção integral aos sujeitos, por meio da comunicação, liderança, tomada de decisão, e gerenciamento de situações e pessoas, de forma crítica e reflexiva^(2)^.

No que diz respeito às legislações da enfermagem dentro da especialidade estética, foi a já mencionada Resolução COFEN nº 529/2016 que abriu o caminho, ao tornar os enfermeiros estetas aptos para realizar carboxiterapia, uso de cosméticos e cosmecêuticos, dermopigmentação, drenagem linfática, eletroterapia/eletrotermofototerapia, terapia combinada de ultrassom e microcorrentes, micropigmentação, ultrassom cavitacional, e vacuoterapia^(1)^.

Não raro, novas intervenções geram conflitos nos limites de atuação entre os profissionais de saúde, e em meio a ordens judiciais públicas, mobilização dos enfermeiros nos eventos científicos e debates pelo reconhecimento profissional na área, a Resolução COFEN n° 529/2016 foi alterada pela Resolução nº 626/2020, a qual assegura a atuação do enfermeiro especialista na área, respeitada a não execução de procedimentos estritos aos profissionais médicos^(1)^.

Em decorrência dos avanços científicos e tecnológicos e demandas do enfermeiro esteta, o Parecer de Câmara Técnica nº 001/2022/GTEE/COFEN adicionou mais técnicas à gama das já permitidas, como plasma rico em plaquetas, aplicação intramuscular de toxina botulínica, endermoterapia, harmonização facial e outras técnicas injetáveis, além de aplicação de fios de sustentação de polidioxanona para bioestimulação por meio de cânulas e indução percutânea de ativos e preenchedores dérmicos^(2)^.

Na sequência, a Resolução nº 715/2023 detalhou o rigor para a legitimidade da especialidade, cuja pós-graduação *lato sensu*, além de devidamente reconhecida pelo Ministério da Educação e Cultura, precisa contar com o mínimo de 100 horas de atividades práticas supervisionadas^(1)^.

Isso posto, diante de tanto tecnicismo, haveria espaço para uma prática que, além de proporcionar cuidados com a aparência, procuraria olhar para o indivíduo além de seu corpo físico, a fim de proporcionar um cuidado em estética mais saudável, menos estigmatizante e mais integral? Intervenções que utilizem recursos de origem natural e que garantam facilidade de acesso, uso e efeitos de longo prazo, juntamente ao movimento de expansão das PICS para a promoção da saúde por meio da ótica da saúde integrativa, com abordagem holística dos indivíduos, colocam a aromaterapia como uma alternativa viável para tratamentos estéticos que procuram integrar saúde aos cuidados em beleza.

### A aromaterapia e a estética

O termo “aromaterapia” foi concebido em 1935 por René Maurice Gattefossé. Em 1937, Gattefossé publicou o livro “*Aromathérapie*”, com a divulgação de um vasto conhecimento científico acerca das relações entre as propriedades das moléculas presentes nos mais diversos OEs, situando a França como um país de forte influência na história da aromaterapia científica^(5)^. Na Inglaterra, inspirada por Gattefossé, a enfermeira Marguerite Maury iniciou os trabalhos de aromaterapia aplicada aos cuidados com a beleza, juventude e bem-estar^(5)^, e em 1961, publicou o livro “*Le Capital Jeunesse*”. A expansão na utilização da aromaterapia conduziu à sua inclusão na Classificação Internacional de Doenças - 11ª revisão no capítulo X - Extensão de códigos, no item de uso pessoal - XE4KX Óleos essenciais, óleos utilizados na aromaterapia.

Com o objetivo de fornecer cuidados para a pele, melhorando seu aspecto e aparência, as formulações em estética têm o objetivo de prevenir e/ou tratar os efeitos do envelhecimento, contando com ativos que retardam ou até mesmo revertam os danos ambientais e da idade. À vista disso, estudos apontam para o potencial dos ativos naturais no combate ao envelhecimento cutâneo, reparando os danos à elastina, atuando contra os radicais livres e o fotoenvelhecimento, e melhorando a hidratação, regeneração e nutrição da pele^(3,4,9)^.

Recursos vegetais apresentam a vantagem de serem considerados mais confiáveis e seguros, de ação suave e eficaz, quando comparados às moléculas sintéticas^(9)^, e têm sido usados há séculos para fins de cuidados com a pele. Na Índia, China, Egito e Irã, confirma-se o uso milenar de plantas com finalidade cosmética^(4)^. Na atualidade, há um aumento progressivo na procura por cosméticos com padrões de produção e de consumo sustentáveis, sendo que, até 2021, cerca de 40 bilhões de dólares já foram gastos em produtos cosméticos de origem natural, cerca de 10% do mercado global do setor^(4)^.

Neste cenário, encontram-se compostos voláteis de baixo peso molecular provenientes das mais diversas partes das plantas (flores, folhas, galhos, sementes, caules, frutos e raízes), formados por uma mistura de substâncias orgânicas, como hidrocarbonetos terpênicos, álcoois (*e.g*., geraniol), aldeídos (*e.g*., citronelal), cetonas e ésteres^(9)^. Assim como outros recursos vegetais, também são milenarmente usufruídos para finalidade estética e, hoje, apresentam ampla relevância nas indústrias farmacêutica, agrícola, alimentícia, cosmética e de saúde^(3,4,9)^.

Propriedades antimicrobianas e antioxidantes, de combate ao estresse oxidativo e aos radicais livres^(3,4,9)^, garantem a presença de OEs em diversas formulações inovadoras para cuidados, limpeza e proteção da pele^(9)^, em crescente substituição aos antioxidantes sintéticos, os quais apresentam efeitos tóxicos para o corpo^(4)^ e danosos ao meio ambiente.

Compostos orgânicos, como o geraniol e o citronelal, encontrados nos óleos de gerânio (*Pelargonium graveolens*) e de rosa-de-damasco (*Rosa x damascena*), atuam na eliminação desses radicais livres e na redução do estresse oxidativo^(5,8)^, o que os tornam aliados promissores para o combate ao envelhecimento cutâneo. Ainda, óleos com diferentes composições bioquímicas, como o pau-rosa (*Aniba roseadora*) e o tomilho qt linalol (*Thymus vulgaris qt linalol*), também possuem propriedades antienvelhecimento cutâneo, ao atenuar rugas e linhas finas^(5)^.

A partir desses dados, é cabível refletir acerca da interlocução, aplicabilidade e potencialidade da aromaterapia na especialidade de enfermagem estética: interlocução, pelo fato de ambas abrangerem um cuidado com efeitos que resultam em bem-estar aos indivíduos em tratamento; aplicabilidade, pelas inúmeras propriedades (dos OEs) antissépticas (*Cinnamomum verum*), anti-inflamatórias (*Lavandula angustifolia*), analgésicas (*Mentha x piperita*) e antioxidantes (*Pelargonium graveolens*)^(5)^, as quais permitiriam, por exemplo, o uso tópico domiciliar de formulações contendo OEs para a maximização de efeitos da intervenção estética executada em consultório, ou o uso concomitante de um OE com ação analgésica para a realização de um procedimento estético doloroso; e potencialidade, pelo uso da aromaterapia como uma prática integrativa e complementar aos procedimentos estéticos usuais, ou ainda respeitadas as preferências dos indivíduos como uma técnica isolada de intervenção mais natural na estética.

Encontrar soluções para tratar os sinais de envelhecimento da pele é, há séculos, objeto de desejo do ser humano, e o mercado da estética está repleto de recursos para essa finalidade. Além disso, a aromaterapia se adequa perfeitamente à política de cosmética sustentável.

Dentro desse cenário, o aumento da procura por produtos de origem natural, com menor impacto na saúde corporal e no meio ambiente, assim como por procedimentos estéticos que sejam menos invasivos, indica um eixo de convergência em ascensão entre a aromaterapia e a estética, ainda que o acentuado senso de urgência com o qual vivemos e nos acostumamos dificulte a aceitação de técnicas que, sendo naturais e não invasivas, possam levar um tempo maior para garantir resultados.

## CONSIDERAÇÕES FINAIS

A partir das reflexões tecidas, finalizar com o incentivo a produções científicas que procurem esmiuçar o entrelaçar dessas duas áreas, de modo a ampliar a oferta de tratamentos estéticos à população interessada e garantir que profissionais enfermeiros estetas tenham cada vez mais subsídios para atuação na área, parece apropriado. As recentes legislações da enfermagem estética no Brasil nos mostram que a especialidade está em evidência e constante desenvolvimento, o que reforça a necessidade de trabalhos que possibilitem a transferência de conhecimentos para uma prática segura e eficaz.

Destaca-se a natureza reflexiva deste trabalho, que procura tecer provocações em torno de intervenções naturais menos invasivas para os cuidados em estética, uma vez que, imersos em uma sociedade repleta de padrões estigmatizantes e adoecedores, e que consome e gera quantidades estarrecedoras de resíduos sintéticos, danosos tanto para a saúde humana quanto para o meio ambiente, é urgente ampliarmos a discussão da sustentabilidade até mesmo para o mercado da estética e, ainda, revermos a forma como enxergamos - ou refutamos - os ideias de beleza.

Todavia, mergulhados em uma realidade imediatista, de ritmo frenético e já muito tomados pelo padrão de beleza universal, inserir no mercado da estética uma prática que, além de somar com aqueles que desejam escancarar uma beleza nem sempre tão simétrica, permite associar o cuidado estético ao cuidado holístico, pode ser além de desafiador para muitos, inaceitável para alguns.

Sobretudo, este trabalho procura despertar oportunidades nas áreas da enfermagem e saúde, e no contexto da aromaterapia aplicada à estética, dado que, além da ampliação do escopo de atuação de enfermeiros e demais profissionais de saúde, como já dito, o contínuo avanço técnico na área da estética não acompanha a acessibilidade de sua oferta de procedimentos para boa parte da população, devido ao custo de determinados protocolos, à sua durabilidade e à não cobertura pela saúde suplementar.

## Data Availability

Não se aplica.
